# MCC950, a selective NLPR3 inflammasome inhibitor, improves neurologic function and survival after cardiac arrest and resuscitation

**DOI:** 10.1186/s12974-020-01933-y

**Published:** 2020-08-31

**Authors:** Maorong Jiang, Ran Li, Jingjun Lyu, Xuan Li, Wei Wang, Zhuoran Wang, Huaxin Sheng, Weiguo Zhang, Jörn Karhausen, Wei Yang

**Affiliations:** 1 Department of Anesthesiology, Center for Perioperative Organ Protection, Duke University Medical Center, Durham, NC USA; 2 Key Laboratory of Neuroregeneration of Jiangsu and Ministry of Education, Co-Innovation Center of Neuroregeneration, Nantong University, Nantong, China; 3 Department of Emergency Medicine, Renmin Hospital of Wuhan University, Wuhan, Hubei China; 4 Department of Immunology, Duke University Medical Center, Durham, NC USA

**Keywords:** Ischemia/reperfusion, NLRP3, Inflammasome, Sterile inflammation, Immunosuppression, Neuroinflammation, CPR

## Abstract

**Background:**

Cardiac arrest (CA) is associated with high morbidity and mortality, even after spontaneous circulation is re-established. This dire situation is partly due to post-CA syndrome for which no specific and effective intervention is available. One key component of post-CA syndrome is sterile inflammation, which affects various organs including the brain. A major effector of sterile inflammation is activated NLRP3 inflammasome, which leads to increased release of interleukin (IL)-1β. However, how NLRP3 inflammasome impacts neuroinflammation and neurologic outcome after CA is largely undefined.

**Methods:**

Mice were subjected to a potassium-based murine CA and cardiopulmonary resuscitation (CPR) model. MCC950 was used to suppress activation of NLRP3 inflammasome after CA/CPR. Levels of protein and mRNA were examined by Western blotting and quantitative PCR, respectively. Immunologic changes were assessed by measuring cytokine expression and immune cell compositions. CA outcomes, including neurologic deficits, bacterial load in the lung, and survival rate, were evaluated.

**Results:**

Using our CA/CPR model, we found that NLRP3 inflammasome was activated in the post-CA brain, and that pro-inflammatory cytokine levels, including IL-1β, were increased. After treatment with MCC950, a potent and selective NLRP3 inflammasome inhibitor, mice exhibited improved functional recovery and survival rate during the 14-day observational period after CA/CPR. In line with these findings, IL-1β mRNA levels in the post-CA brain were significantly suppressed after MCC950 treatment. Interestingly, we also found that in MCC950- vs. vehicle-treated CA mice, immune homeostasis in the spleen was better preserved and bacterial load in the lung was significantly reduced.

**Conclusions:**

Our data demonstrate that activation of NLRP3 inflammasome could be a key event shaping the post-CA immuno- and neuro-pathology, and identify this pathway as a unique and promising therapeutic target to improve outcomes after CA/CPR.

## Background

Cardiac arrest (CA) is a devastating medical emergency [1]. Currently, the only specific treatment is early resuscitation. Still, even successful resuscitation may not ensure a good prognosis, as a substantial number of CA patients with return of spontaneous circulation (ROSC) either do not survive to hospital discharge or live with lifelong disabilities. Given that resuscitation techniques are continuously optimized and the likelihood of surviving a CA event is increasing, there is an urgent need to improve post-resuscitation treatment to better serve this growing population of CA survivors.

Mounting evidence has attributed a critical importance to the immune response of the post-CA syndrome in determining overall survival as well as neurologic recovery [2–5]. However, how the post-CA immune response is regulated and consequently, how to intervene to ameliorate its detrimental effects remain largely unknown. CA and resuscitation causes global ischemia/reperfusion insult, a well-established event triggering a sterile inflammatory response that may eventually lead to tissue damage [6, 7]. Initiation of sterile inflammation is mediated through activation of common pattern recognition receptors (PRRs) that can detect host-derived (self) damage-associated molecular patterns (DAMPs) released by damaged or dying cells. Importantly, various DAMPs have been detected in the blood of CA patients in the first days after cardiopulmonary resuscitation (CPR) [8]. Thus, sterile inflammation after CA and resuscitation is a key element in the CA pathophysiology.

Inflammasomes have emerged as central effectors of sterile inflammation mounted by the innate immune system. In particular, the NOD-like receptor (NLR) family, pyrin domain-containing protein 3 (NLRP3) inflammasome, plays a critical role in many diseases involving sterile inflammation, especially ischemia-related diseases such as ischemic stroke, acute kidney injury, intestinal ischemia, and lung ischemia [9]. NLRP3 inflammasome consists of a cytoplasmic PRR (i.e., NLRP3), an adaptor protein known as ASC (apoptosis-associated speck-like protein containing a CARD), and an effector enzyme (caspase-1). In response to DAMPs, NLRP3 inflammasome is activated via a 2-step process—priming and activation. The priming step serves to upregulate expression of the inflammasome components, including NLRP3 and caspase-1. In the activation step, NLRP3 oligomerization and subsequent recruitment of ASC and caspase-1 occurs, and an intracellular multiprotein complex, i.e., inflammasome, is assembled, leading to activation of caspase-1. Activated caspase-1 then cleaves pro-interleukin-1β (pro-IL-1β) and pro-IL-18 to generate bioactive IL-1β and IL-18, which are secreted from the cell to exert potent inflammatory effects in the tissue locally or the circulation systemically. Notably, IL-1β is a master cytokine that can initiate and amplify the innate immune response and inflammation, and thus is a primary pathologic contributor in a wide array of diseases [10]. Since activation of NLRP3 inflammasome is a major mechanism by which cells produce IL-1β, it is not surprising that NLRP3 has been implicated in the pathogenesis of numerous diseases involving various organs including lung [11], liver [12], kidney [13], heart [14], and brain [15].

After CA, IL-1β is markedly increased in the circulation and brain, strongly indicating the involvement of NLRP3 inflammasome in the post-CA immune response [5, 16, 17]. Yet, the role of NLRP3 inflammasome in CA has not been well-established. Understanding its role may lead to development of new immunomodulatory therapeutics to improve CA outcome. Thus, in this study, we used our clinically relevant mouse model of CA/CPR to investigate the role of NLRP3 inflammasome in CA/CPR with a focus on the brain and neurologic outcome because CA-induced brain damage is a primary factor that determines post-CA mortality and morbidity. MCC950, a selective NLRP3 inhibitor, was used to suppress activation of NLRP3 inflammasome [18].

## Methods

### Animals

All animal experiments were conducted according to the guidelines of Duke University and were approved by the Institutional Animal Care and Use Committee. Male C57Bl/6 mice (3–4 months old) were obtained from Jackson Laboratory, and kept in a room at constant temperature and humidity and a light/dark cycle of 14:10 h (3–5/cage). The online tool Quickcalcs was used to randomize animals for group assignments.

### Animal surgeries

CA/CPR surgery was performed essentially as previously described [19]. Briefly, after oral intubation, mice were maintained on 1.5–1.7% isoflurane and at 37 ± 0.2 °C (rectal probe) before CA onset. Electrocardiogram (ECG) and peripheral blood flow were monitored throughout the procedure. Immediately after CA induction by potassium chloride (KCl) injection, the ventilator and body temperature control system were turned off, while brain temperature was maintained at 38.5 ± 0.2 °C using a coil with circling warm water. At 8.5 min CA, the brain temperature control system was turned off, mechanical ventilation with 100% O_2_ was resumed, and a bolus of epinephrine (100 μL of 32 μg/mL) was administered followed by continuous infusion of epinephrine (25 μL/min). Chest compression was performed until return of spontaneous circulation (ROSC), defined as return of stable ECG sinus rhythms. If ROSC could not be achieved within 3 min, resuscitation was discontinued, and the animal was excluded from the study.

Ischemic stroke surgery was performed as described previously [20]. Briefly, mice were orally intubated and mechanically ventilated with 1.5–1.7% isoflurane. Throughout the whole procedure, the rectal temperature was maintained at 37 ± 0.2 °C, and cerebral blood flow was monitored using Laser-Doppler flowmetry (Moor Instruments). Transient middle cerebral artery occlusion (MCAO) was achieved by inserting a monofilament (Doccol Corp, Sharon, MA, USA) into the internal carotid artery via the external carotid artery and temporary ligation of the right common carotid artery with suture. After 45 min of ischemia, perfusion was restored by removing the filament and the suture.

### Drug administration

MCC950 (Sigma-Aldrich, St. Louis, MO, USA) was dissolved in phosphate-buffered saline (PBS). Mice were administered MCC950 (10 mg/kg) or PBS (vehicle) intraperitoneally (ip) daily for 3 consecutive days starting at 15 min post ROSC.

### Behavioral tests

All behavioral tests were performed during the light phase by experimenters who were blinded to group assignments.

#### Neurologic score

A 9-point scoring system was used to assess overall neurologic deficits [19]. The final score ranks from 0 to 9 (9 points = normal and 0 point = severe injury).

#### Rotarod

Rotarod was conducted as previously described [19]. Mice were gently placed on the rotarod, and within 5 min, the rotation speed was gradually increased to 40 rpm. When mice fell off the rotarod, a trial ended, and the time lapse on the rotarod was recorded. Animals were trained for 3 days before CA/CPR surgery. Data are presented as mean duration (3 trials) on the rotarod.

#### Open field test

On the day of the test, mice were transferred to the testing room and left in their home cages for 1 h before the test. To start each session, the mouse was placed in the center of the open field box (50 × 50 × 50 cm, CleverSys Inc, Reston, VA, USA), and allowed to explore for 10 min while being recorded by an overhead camera. The video clips were then analyzed by the automated tracking system TopScan (CleverSys).

#### Object location memory test

The object location memory test was performed as described previously with minor modifications [21]. In brief, before testing, all mice were gently handled for 2–3 min by the experimenter, and were habituated to the testing environment (without objects) for 10 min each day for 2 consecutive days. During the training phase, 2 identical objects were placed in the open field box (50 × 50 × 50 cm), and mice were allowed to explore the objects for 10 min. Twenty-four hours after the training, one object was relocated to a new position in the box, and mice were put into the box and observed for 5 min. An exploring event was defined as the mouse nose within 2 cm of an object. The movement was analyzed by the tracking system TopScan (CleverSys) for the following parameters: time spent in exploring the object moved to a new position, time spent in exploring the object remaining in the familiar place, and total time spent in object exploration. Percentage of time spent exploring object in new position = time exploring the object in the new position/(total time spent in object exploration) × 100.

### Quantitative reverse transcription–polymerase chain reaction

Standard procedures were applied [19]. In short, total RNA was prepared from brain cortex tissues using TRIzol reagent (Invitrogen, Carlsbad, CA, USA), and was then used to generate cDNA samples. Quantitative PCR was performed using a QuantStudio 3 (ThermoFisher Scientific, Waltham, MA, USA). All primers used in this study are listed in supplemental Table S1.

### Western Blotting

Our standard method was used for Western blotting [19]. Brain cortex tissues were quickly dissected out on ice, and homogenized by sonication using lysis buffer supplemented with 2% SDS. Primary antibodies used are listed in supplemental Table S2. Quantification of signal intensities was performed using ImageJ (NIH, Bethesda, MD, USA).

### Preparation of single cell suspensions for flow cytometry

Cell preparation was performed essentially as previously described [5]. Spleen tissues were gently pressed to pass through 70-μm cell strainers to obtain single cell suspensions, followed by red blood cell lysis. Brain tissues were cut into small pieces with scissors in ice-cold DMEM medium, and digested with Collagenase D (1 mg/mL) and DNAse I (1 mg/mL) (Sigma-Aldrich, St. Louis, MO, USA) for 45 min at 37 °C. Cells were then filtered through a 70-μm cell strainer, and resuspended in a 70%/37% Percoll gradient (GE Healthcare Life Sciences, Pittsburgh, PA, USA). After centrifugation at 500×*g*, the immune cells were harvested at interphase. After washing, all isolated cells were resuspended in 1 mL PBS containing 1% fetal calf serum, and stained with Trypan blue to count with a hemocytometer in triplicate.

### Flow cytometry analysis

Standard procedures were followed [5]. Briefly, single cell suspensions were adjusted to approximately 1 × 10^6^ cells in 100 μL DMEM containing 2% fetal calf serum. After incubation with Fc receptor blocking solution for 15 min, leukocyte subpopulations were immunostained with combinations of different surface antibodies (listed in supplemental Table S2). Flow cytometry data were acquired on FACS Canto (BD Biosciences, San Jose, CA, USA) and analyzed using FlowJo software.

### Blood-brain barrier (BBB) permeability

The integrity of the BBB was evaluated using Evans blue (EB) extravasation. EB (2% in saline, 100 μL per mouse; Sigma-Aldrich) was injected intravenously via the tail vein after stroke or CA/CPR. After 3 h or 24 h reperfusion, mice were anesthetized and transcardially perfused with saline. Brains were removed and coronally sliced using a mouse brain matrix (ASI Instruments, Warren, MI). Brain sections (1 mm thick) were then fixed in 10% formaldehyde.

### Bacteriologic analysis

Bacteriologic analysis was performed as previously described [5]. On day 3 after CA/CPR, lung tissues were dissected under sterile conditions, and homogenized with 1 mL PBS. The tissue homogenate was serially diluted and cultured on the blood agar plates at 37 °C for 18 h. Bacterial colonies were then counted.

#### Statistical analysis

Data were analyzed using Prism 8 software (GraphPad, La Jolla, CA, USA). The group sizes for each experiment were determined on the basis of our previous studies or pilot experiments. The unpaired Student’s *t* test (all data except neurologic scores) or Mann-Whitney *U* test (neurologic scores) was used to compare 2 groups. The survival curves were analyzed using the Gehan-Breslow-Wilcoxon test. To compare more than 2 groups, one-way ANOVA with post hoc Holm-Sidak correction for multiple comparisons was performed. Data are presented as mean ± SEM. The level of significance was set at *p* < 0.05.

## Results

### NLRP3 inflammasome is activated in the brain after CA/CPR

**Fig. 1:**
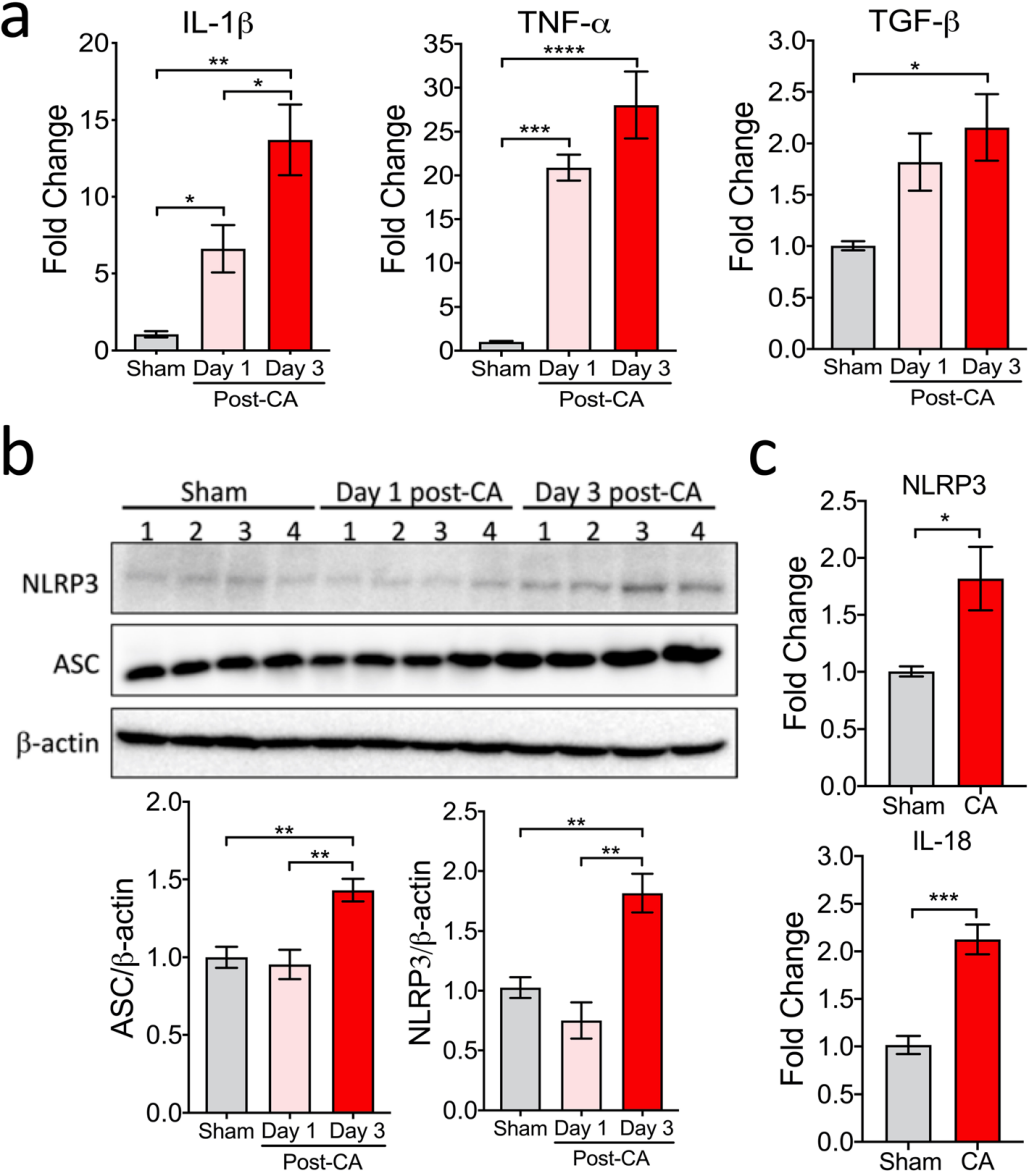
Levels of cytokines and NLRP3 inflammasome components in the brain after CA/CPR. Mice were subjected to sham or CA/CPR surgery. Brain cortical samples were collected on day 1 or day 3 after CA/CPR. **a** Cytokines in the post-CA brain. The mRNA levels of tumor necrosis factor alpha (TNF-α), interleukin 1beta (IL-1β), and transforming growth factor beta (TGF-β), were measured by qRT-PCR. **b, c** NLRP3 inflammasome components in the post-CA brain. **b** The protein levels of NLRP3 and ASC were evaluated by Western blotting. β-actin was used as a loading control. **c** The mRNA levels of NLRP3 and IL-18 in the brain on day 3 after CA/CPR. The mean values in sham samples were set to 1.0. Data are presented as mean ± SEM (*n* = 4/group). *p < 0.05; **p < 0.01; ***p < 0.001

Following CA/CPR, a systemic inflammatory response occurs, with a marked increase in pro-inflammatory cytokine levels in the circulation as well as in the brain [5]. To confirm and expand this finding in the post-CA brain, we first performed a time-course analysis of mRNA levels of cytokines (IL-1β, TNF-α, and TGF-β), using brain samples collected on day 1 and day 3 after CA/CPR. As expected, mRNA levels of all 3 cytokines were already upregulated in the brain on day 1, and remained elevated on day 3 after CA/CPR (Fig. 1a). Notably, in contrast to TNF-α and TGF-β, IL-1β mRNA levels continued to significantly increase in the brain from day 1 to day 3 post-CA (6.6 ± 1.5 vs. 13.7 ± 2.3; *p* < 0.05), indicating augmented transcriptional upregulation over time.

**Fig. 2:**
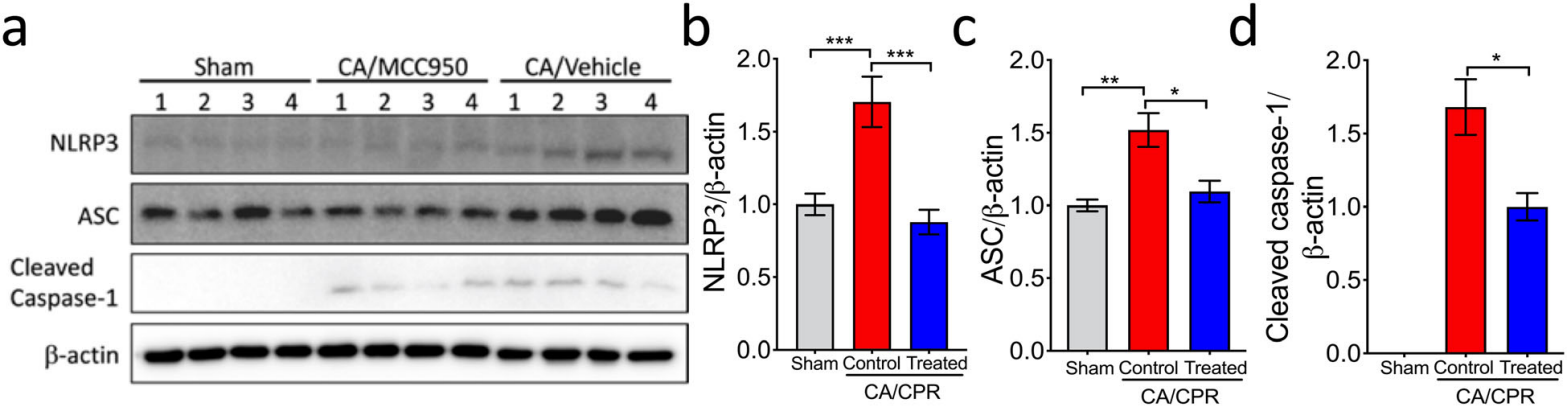
Post-CA activation of NLRP3 inflammasome was suppressed in the brain of MCC950-treated mice. **a** Mice were subjected to sham or CA/CPR surgery. Mice received intraperitoneal injections of MCC950 (treated) or vehicle (control) daily for 3 consecutive days starting at 15 minutes after resuscitation (day 0). On day 3 post-CA, cortex samples were collected for Western blotting analysis. **b–d** Quantification of Western blotting. Intensities of each band were measured and normalized to β-actin. The mean values in sham samples were set to 1.0 (except for cleaved caspase-1). Data are presented as mean ± SEM (*n* = 4/group). *p < 0.05; **p < 0.01; ***p < 0.001

To determine the association between increased IL-1β and NLRP3 inflammasome, we then examined post-CA changes in protein levels of the NLRP3 inflammasome components, NLRP3 and ASC, in the brain by Western blotting in our model. While no change was observed on day 1, the protein levels were significantly higher on day 3 after CA/CPR, compared to sham mice (Fig. 1b). In addition, the mRNA levels of NLRP3 and IL-18 were significantly upregulated in the brain on day 3 after CA/CPR (Fig. 1c). Moreover, the levels of cleaved caspase-1 (i.e., activated caspase-1) dramatically increased on day 3 after CA/CPR (Fig. 2a; vehicle-treated samples), in line with a previous report [16]. Taken together, these data indicated that after CA/CPR, NLRP3 inflammasome was activated and may thus contribute to sustained IL-1β production in the brain.

### Inhibition of NLRP3 inflammasome with MCC950 improves functional outcome after CA/CPR

We then hypothesized that suppression of NLRP3 inflammasome activation in the post-CA brain improves neurologic functional recovery after CA/CPR. We chose MCC950 to test this hypothesis because MCC950 is a potent and specific NLRP3 inflammasome inhibitor. First, we evaluated the effects of MCC950 treatment on NLRP3 inflammasome activation in the post-CA brain. After CA/CPR, mice were dosed with MCC950 or vehicle for 3 days starting at 15 min after reperfusion (day 0). On day 3 post CA, brain samples were collected for Western blotting (Fig. 2). Compared to the sham group, protein levels of NLRP3, ASC, and cleaved caspase-1 were significantly increased in vehicle-treated CA mice, but these increases were largely suppressed by MCC950, confirming its potent inhibitory effect on post-CA NLRP3 inflammasome activation (Fig. 2).

**Fig. 3:**
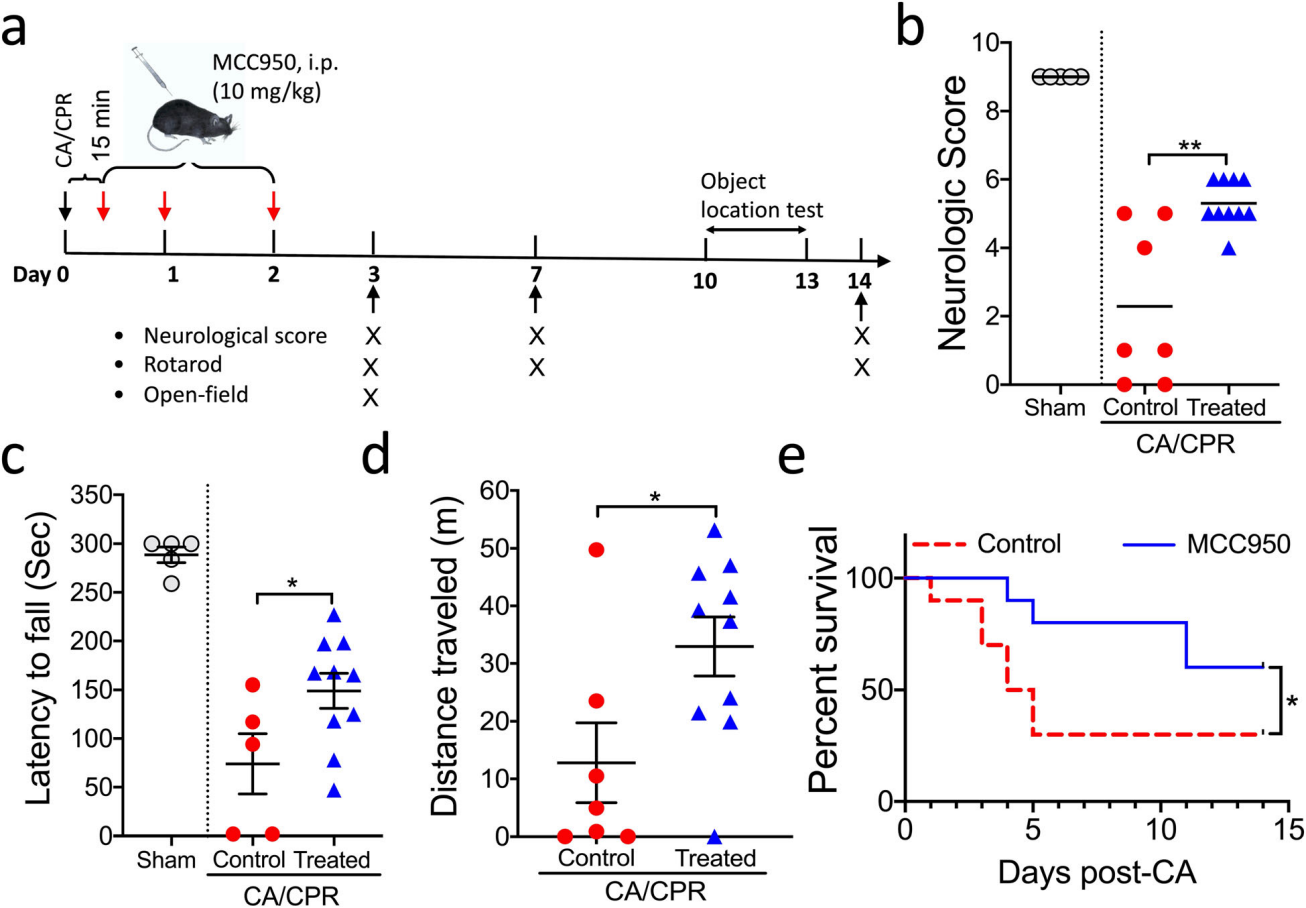
Functional outcome after CA/CPR was improved in MCC950-treated mice. **a** Schematic diagram of the experimental design. Mice were subjected to CA/CPR or sham surgery. After 15-min reperfusion, mice received intraperitoneal (ip) injection of MCC950 (treated) or vehicle (control), followed by daily injection for 2 days. Behavioral tests were performed at the indicated time points. Data from sham-operated mice served as baseline references. **b–d** Functional outcome assessment on day 3 after CA/CPR included **b** neurologic scores, **c** rotarod test (two mice from the vehicle group were not subjected to this test due to severe sickness), and **d** open field test. **e** Survival rate. Data are presented as median or mean ± SEM (*n* = 5–10/group). *p < 0.05; **p < 0.01

Since it is of high clinical relevance to evaluate long-term effects on CA outcome for a pharmacologic intervention, we designed our MCC950 treatment experiment in which behavioral tests were performed up to 14 days after CA/CPR, as shown in Fig. 3a. We also included a sham group (*n* = 5) as a baseline reference. At the first time point for behavioral tests (post-CA day 3), we observed a mortality rate of 30% (3 out of 10) in the vehicle group; in contrast, no mouse died in the MCC950 group. Compared to the vehicle-treated CA mice, MCC950-treated CA mice exhibited significantly better performance on all tests, i.e., neurologic scoring (Fig. 3b), rotarod (Fig. 3c), and open field test (Fig. 3d). After this sub-acute functional evaluation, more mice from both groups died, as expected from this severe disease. Particularly, at the second (post-CA day 7) and third (post-CA day 14) test time points, only 3 out of 10 mice survived in the vehicle group. Despite that the sample size was considered too small for further behavioral tests, we decided to continue the experiment as planned as this situation is intrinsically related to this disease model. Given the potential survival bias, we were not surprised to find no significant difference in the neurologic score and rotarod test between the vehicle and treatment groups (Fig. S1A,B). Interestingly, on day 14 post CA, MCC950-treated mice showed improved memory function on the object location test, which assesses hippocampus-dependent memory function (Fig. S1C). Finally, the overall survival rates of the vehicle- and MCC950-treated groups over the 14 days after CA/CPR were 30% and 60%, respectively (*p* = 0.04; Gehan-Breslow-Wilcoxon test). Collectively, these data demonstrated that post-CA treatment with MCC950 to suppress NLRP3 inflammasome activation improved functional recovery and the survival rate after CA/CPR.

### Inflammatory response in the brain after CA/CPR is attenuated in MCC950-treated mice

**Fig. 4:**
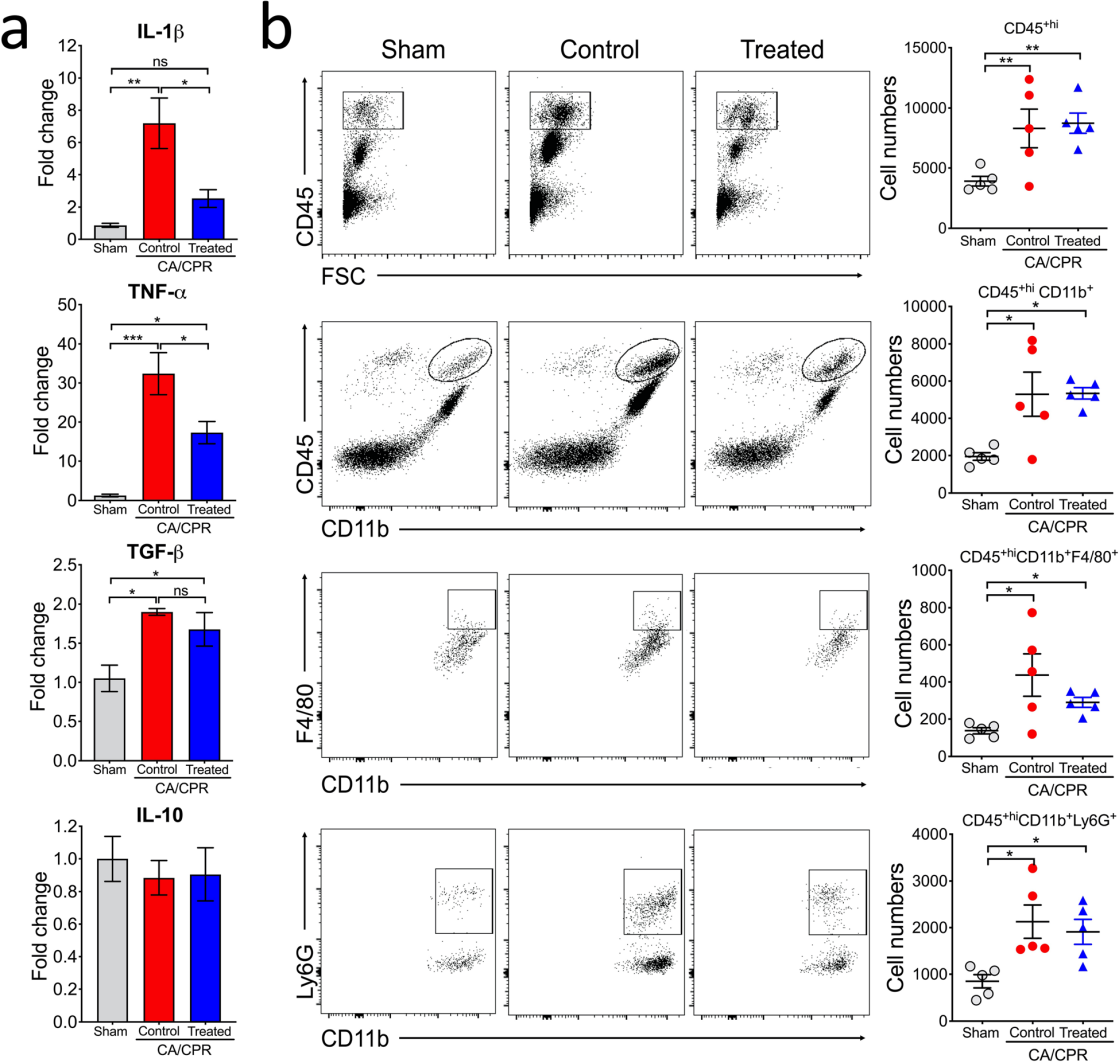
CA-induced inflammatory cytokine expression in the brain was attenuated in MCC950-treated mice. Mice were subjected to sham or CA/CPR surgery. After 15-min reperfusion, mice received intraperitoneal injection of MCC950 (treated) or vehicle (control), followed by daily injection for 2 days. On day 3 post-CA, brains were collected for qRT-PCR and flow cytometry analyses. **a** Expression of cytokines. The mRNA levels (*n* = 4/group) of IL-1β, TNF-α, TGF-β, and IL-10 were measured by qRT-PCR. **b** Leukocyte infiltration into the post-CA brain. Single-cell suspensions were prepared from whole brains and subjected to flow cytometric analysis (*n* = 5/group). Infiltrating leukocytes (CD45^+hi^) and CD45^+hi^CD11b^+^ cells with their subsets, monocytes/macrophages (CD45^+hi^CD11b^+^F4/80^+^), and neutrophils (CD45^+hi^CD11b^+^Ly6G^+^) are quantified and the representative dot plots are also shown here. Data are presented as mean ± SEM. *p < 0.05; **p < 0.01; ***p < 0.001; *ns* not significant

The beneficial effects of MCC950 on neurologic functions after CA/CPR are likely due to its ability to reduce neuroinflammation in the post-CA brain. To test this, we analyzed the mRNA levels of inflammatory cytokines in the brain by quantitative reverse transcription–polymerase chain reaction (qRT-PCR). Consistently, expression levels of IL-1β, TNF-α, and TGF-β were markedly increased in the post-CA brain on day 3 after CA/CPR (Fig. 4a). The increases in IL-1β and TNF-α, but not TGF-β, were significantly suppressed in the MCC950- vs. vehicle-treated CA mice. In line with MCC950 being a specific inhibitor of NLRP3 inflammasome, CA-induced increase in IL-1β expression was almost completely nullified, suggesting the predominant role of NLRP3 inflammasome activation in upregulating IL-1β in the brain on day 3 after CA/CPR. We also examined the anti-inflammatory cytokine IL-10, but the mRNA levels of this cytokine appeared to be unaltered in the post-CA brain (Fig. 4a).

In addition to cytokine upregulation, infiltration of peripheral leukocytes contributes to neuroinflammation in the post-CA brain [5]. Thus, we investigated the effects of MCC950 treatment on infiltrating leukocytes. We found that, consistent with our previous data, the cell number of infiltrating leukocytes (CD45^+hi^) was significantly increased in the brain on day 3 post CA, and that most of these cells were monocytes/macrophages and neutrophils, identified as the CD45^+hi^CD11b^+^ population (Fig. 4b). We did not detect any change in T and B cells in post-CA brains (Fig. S2), which is in contrast to a significant increase of both cell types in the brain after ischemic stroke [22]. One possible explanation is that BBB disruption was not as severe after global brain ischemia caused by CA/CPR as after focal brain ischemia (i.e., ischemic stroke) in neither the acute (3-h) nor delayed (24-h) phase (Fig. S3). Notably, MCC950 appeared to have no obvious effect on reducing the infiltration of peripheral immune cells into the post-CA brain (Fig. 4b and S2).

### Immune homeostasis is better preserved after CA/CPR in MCC950-treated mice

**Fig. 5:**
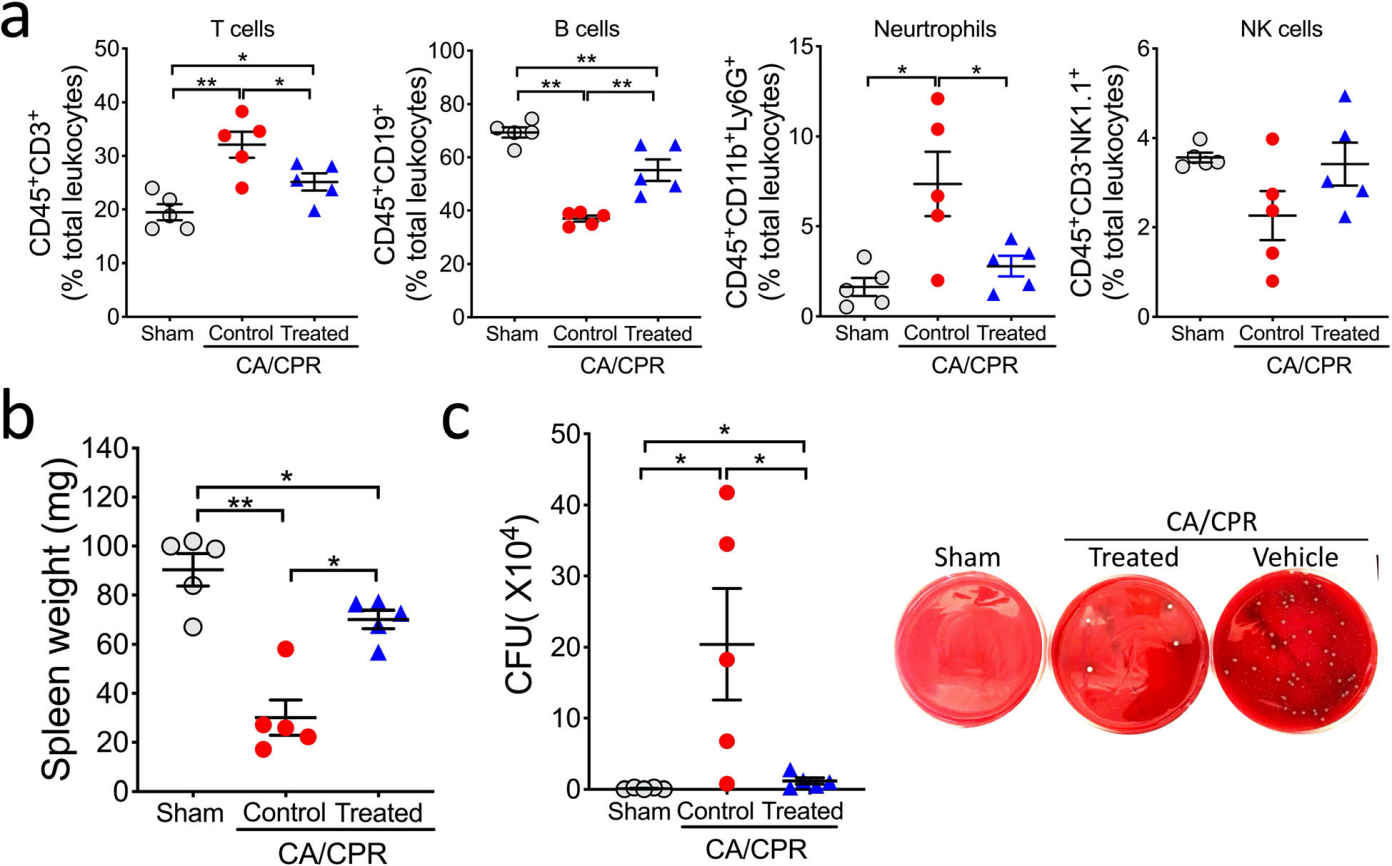
Post-CA immune homeostasis and bacterial load in the lung in MCC950-treated mice. Mice were subjected to sham or CA/CPR surgery. After 15 minutes reperfusion, mice received intraperitoneal injection of MCC950 (treated) or vehicle (control), followed by daily injection for 2 days. All analyses were performed on day 3 after CA/CPR. **a** Immune cell populations in the spleen. The following leukocyte subpopulations are shown here: T cells (CD45^+^CD3^+^), B cells (CD45^+^ CD19^+^), neutrophils (CD45^+^CD11b^+^Ly6G^+^), and natural killer cells (NK; CD45^+^CD3^-^NK1.1^+^). **b** Spleen weight. **c** Bacterial load in the lung. Representative images illustrate the colonies on blood agar plates. Data are presented as mean ± SEM (*n* = 5/group). *p < 0.05; **p < 0.01; ***p < 0.001

Besides neuroinflammation in the post-CA brain, CA/CPR also disrupts immune homeostasis in the peripheral organs, leading to a complex and dysregulated immunologic response, especially profound suppression of the immune organs at a late stage, as identified recently [5]. We thus wanted to know whether MCC950 treatment has any effect on post-CA defects in immune organs. To this end, we examined the spleen. We have showed previously that on day 3 after CA/CPR, the percentages of T cells and neutrophils in the spleen are increased while the percentage of B cells is markedly decreased [5]. Similar changes were observed here in vehicle-treated CA mice (Fig. S4 and Fig. 5a). These changes—representing homeostatic imbalance of the immune system caused by CA/CPR—were significantly mitigated in MCC950-treated mice. Notably, MCC950 treatment also partially prevented CA-induced splenic atrophy (Fig. 5b), a major phenotype of post-CA immunosuppression [5]. Collectively, MCC950-treated CA mice exhibited improved immune homeostasis compared to vehicle-treated CA mice. Based on these data, we expected that the CA-related increase in bacterial load in the lung would be less in MCC950-treated vs. control mice. Indeed, as shown in Fig. 5c, the number of bacterial colonies cultured from homogenized lung was robustly increased in the CA group compared to the sham group; however, this increase was significantly reduced in MCC950-treated mice.

## Discussion

There is an urgent need for an effective treatment to improve prognosis for CA patients who are successfully resuscitated. The dysregulated immune response after CA/CPR has been proposed as a major defining factor for progression of post-CA syndrome and as such, a primary therapeutic target for post-CA treatment. In this study, we provided evidence that the CA-induced increase in pro-inflammatory IL-1β in the brain is largely mediated by NLRP3 inflammasomes. More importantly, pharmacologic inhibition of NLRP3 activation using the small molecule MCC950 after CA/CPR significantly attenuated neuroinflammation in the brain, preserved immune homeostasis, reduced overall mortality, and improved neurologic outcome.

At least 5 PPRs can initiate assembly of inflammasomes. These include NLRP1, NLRP3, NLRC4, ALR, and AIM2. Inflammasomes assembled by these PRRs have the similar multiprotein structure, and all lead to activation of caspase-1 and the subsequent maturation and secretion of the inflammatory cytokine IL-1β. Although the current study focused on NLRP3 inflammasome, other inflammasomes may also be involved in the increase of IL-1β in the post-CA brain. Indeed, our time-course data showed that after CA/CPR, NLRP3 protein levels in the brain were increased on day 3 but not on day 1 when IL-1β mRNA levels were already increased, although to a lesser extent than on day 3. This finding implies that the early post-CA increase in IL-1β may result from activation of inflammasomes other than NLRP3. Such a notion is supported by the unique activation process of NLRP3 inflammasome, which requires a priming step to transcriptionally upregulate expression of the components including NLRP3, followed by a second step to assemble the active inflammasome [23]. In comparison, activation of other inflammasomes does not require this delay time for mRNA transcription and following protein translation. Thus, our data may indicate the critical involvement of the NLRP3 inflammasome in the sub-acute phase after CA/CPR.

NLRP3 inflammasome can be activated by a large number of highly diverse structures of DAMPs as well as pathogen-associated molecular patterns (PAMPs) [9]. However, the exact mechanisms that underpin NLRP3 activation by these multiple triggers are not well characterized. Given the broad spectrum of activators for NLRP3 inflammasome, it is unlikely that NLRP3 interacts physically with a given activator. In fact, it is now widely believed that unlike other inflammasome PPRs, NLRP3 reacts to intracellular damage or stress signals induced by relevant activators. Such signals include K^+^ efflux, production of reactive oxygen species (ROS), and Ca^2+^ mobilization. By testing various NLRP3 activators, one study provided strong evidence that K^+^ efflux is a common signal that is sufficient to activate NLRP3 inflammasome [24]. Another group reported that ROS engages NLRP3 inflammasome by regulating the priming step [25]. Murakami et al. found that Ca^2+^ mobilization, e.g., Ca^2+^ release from endoplasmic reticulum (ER), is a critical upstream signal in NLRP3 inflammasome activation, and more specifically, that ER stress, which leads to the release of Ca^2+^ from the ER lumen, amplifies NLRP3 inflammasome activation [26]. Importantly, all these signals are activated after brain ischemia [19, 27, 28], which likely underlies activation of NLRP3 inflammasome in the brain after CA/CPR, as observed here. Future research is required to determine the extent to which these signals contribute to NLRP3 inflammasome activation after CA/CPR.

The current study directly examined the role of NLPR3 inflammasome in CA/CPR with a focus on the brain. However, CA/CPR imposes ischemia/reperfusion insult on all organs. Thus, it is plausible to assume that NLPR3 inflammasome also plays a critical role in mediating tissue damage in other organs. Indeed, many studies have used organ-specific ischemia/reperfusion models to investigate NLPR3 in individual organs. For example, after ischemic acute kidney injury, NLRP3 expression is increased, and NLRP3^−/−^ vs. wild-type mice exhibit significantly lower acute tubular necrosis and apoptosis scores in the kidney [29]. Similarly, lung ischemia/reperfusion injury triggers activation of NLRP3 inflammasome, which is responsible for inflammation-induced lung injury [30]. Further, a large body of evidence underscores the critical contribution of NLRP3 inflammasome in acute myocardial infarction and its consequences on adverse cardiac remodeling and heart failure [14]. Finally, several studies showed that pharmacologic or genetic deletion or inhibition of NLRP3 reduces infarct sizes and improves neurologic function after ischemic stroke [31, 32]. Taken together, it is reasonable to propose that a concomitant immune response after ischemia/reperfusion injury in an organ is to activate NLRP3 inflammasome, which may then exert a detrimental role in inflammation-mediated tissue injury. Therefore, NLRP3 inflammasome appears to be a unique, promising therapeutic target for CA/CPR, which causes whole-body ischemia/reperfusion and affects all organs.

Notably, in addition to ischemia/reperfusion diseases, inflammasomes have been implicated in the pathogenesis of many other diseases, including neurodegenerative diseases. Thus, development of small molecules that target different inflammasomes, but primarily NLRP3 inflammasome, has attracted considerable attention over the past decades [10, 33]. MCC950 is regarded as the most specific NLRP3 inhibitor to date, as it does not inhibit the NLRP1, NLRC4, and AIM2 inflammasomes [18]. Its molecular mechanism of action was recently revealed. MCC950 binds to NLRP3, blocks its ability to hydrolyze ATP, and thus prevents it from maintaining its active structural conformation, thereby inhibiting NLRP3-induced ASC oligomerization and reducing cleavage of caspase-1 [18, 34]. Indeed, we found that MCC950 treatment reduced the protein levels of cleaved caspase-1 in the post-CA brain. Interestingly, we also noted that CA-induced protein levels of NLRP3 and ASC via the priming step were significantly suppressed by MCC950. This may not be unexpected, since previous studies have reported a similar finding [35]. Although we do not yet know how MCC950 affects the priming step of NLRP3 activation, a positive feedback loop mechanism induced by NLRP3 inflammasome itself may account for this effect [36]. Nevertheless, MCC950 has been tested in various experimental models of diseases including Alzheimer’s disease [37], traumatic brain injury [38], and stroke [35]. Data from these studies predominantly attest to the beneficial effects of MCC950 in these diseases in which an inflammatory response is central to their pathogenesis. Our data lend further support to this notion, as we provided evidence that MCC950 treatment improves CA outcome.

Understanding the role of inflammasomes in CA has just began. The current study focused on NLRP3 inflammasome in the brain after CA/CPR, but many interesting questions remain to be explored. For example, activation of NLRP3 inflammasome can lead to a pro-inflammatory form of cell death known as pyroptosis. Key to this programmed cell death process is the pore-forming protein gasdermin D (GSDMD) [39]. Whether GSDMD-mediated pyroptosis is critically involved in CA-induced cell death needs to be clarified. Further, CA/CPR causes inflammation in multiple organs, which at least partly contributes to eventual organ failure. The extent to which inflammasomes are responsible for inflammatory injury in different organs after CA/CPR is almost completely unknown. Finally, as discussed above, inflammasomes other than NLRP3 inflammasome may be involved in the pathophysiology of CA/CPR. Indeed, NLRC4 and AIM2 inflammasomes have been implicated in brain ischemia, as a study showed that after transient ischemic stroke, mice with global deletion of *Nlrc4* or *Aim2* have smaller infarct volumes and better neurologic scores compared to wild-type mice [40]. Of note, AIM2 is a cytosolic PRR that recognizes double-stranded DNA including self-DNA from nuclei or mitochondria [39]. Clearly, more research is warranted in this field.

Some limitations in the current study were noted. Although we examined several key components of the NLRP3 inflammasome pathway in the post-CA brain, detailed characterization of NLRP3 activation over time after CA/CPR is still lacking. For example, future studies with a longer observational period are needed to fully define the timeline and dynamics of post-CA NLRP3 inflammasome activation by evaluating major genes involved in NLRP3 inflammasome at both mRNA and protein levels, and also the ASC speck formation—an indicator of NLRP3 activation. Moreover, to further explore the therapeutic potential of targeting NLRP3 inflammasome, optimized CA/CPR surgical conditions are required to minimize potential survival bias for long-term CA outcome research in both young and aged animals.

## Conclusions

NLRP3 inflammasome may represent a potential novel target for new therapeutics that reduce sterile inflammation-related tissue damage and preserve immune homeostasis, thus improving post-resuscitation prognosis for CA survivors.

## Supplementary information

**Additional file 1: Figure S1.** Long-term outcome after CA/CPR in MCC950-treated mice. Supplemental data to Fig. 3. Mice were subjected to sham or CA/CPR surgery. After 15 minutes reperfusion, mice received intraperitoneal injection of MCC950 (10 mg/kg) or vehicle, followed by daily injection for 2 days. Rotarod and neurologic scoring were performed (A) on day 7 and (B) on day 14. The object location memory test was performed (C) from day 10 to 13. A schematic (right) illustrates 3 trial phases in the object location memory test. Data are presented as mean ± SEM (n = 3-8/group). **, *p* < 0.05; **, *p* < 0.01; ***, *p* < 0.001. **Figure S2.** Flow cytometric analysis of immune cells in the post-CA brain. Supplemental data to Fig. 4. A) The main flow cytometric gating strategy for infiltrating monocytes/macrophages and neutrophils. B) Mice were subjected to sham or CA/CPR surgery. After 15 minutes reperfusion, mice received intraperitoneal injection of MCC950 or vehicle, followed by daily injection for 2 days. On day 3 after CA/CPR, brains were collected for flow cytometry. Quantification of infiltrating leukocyte subsets of T (CD45+hiCD3+), B (CD45+hiCD3-CD19+), and NK (CD45+hiCD3-NK1.1+) cells is shown. Data are presented as mean ± SEM (n = 5/group). **Figure S3.** Disruption of blood-brain barrier (BBB) is not obviously evidenced in the post-CA brain. Mice were subjected to 45 minutes middle cerebral artery occlusion (MCAO) or 8.5 minutes CA. Extravasation of Evans blue (blue color) was evaluated at 3 hours or 24 hours after reperfusion. While BBB breakdown was clearly shown in brains after MCAO, there was no obvious BBB damage in the post-CA brains, indicating a comparably minor effect of CA/CPR on BBB permeability in our CA/CPR model. Representative images are shown from 2 independent experiments. **Figure S4.** The main gating strategy for analyzing immune cell popoluations in the spleen. **Table S1.** Primer sequences. **Table S2.** Antibodies for Western blotting and flow cytometry analysis.

## Data Availability

All data generated or analyzed during this study are included in this published article and its supplementary information files.

## Abbreviations

ASC: Apoptosis-associated speck-like protein containing a CARD
CA: Cardiac arrest
CPR: Cardiopulmonary resuscitation
DAMPs: Damage-associated molecular patterns
EB: Evans blue
ECG: Electrocardiogram
ER: Endoplasmic reticulum
IL-1β: Interleukin-1β
MCAO: Middle cerebral artery occlusion
NLRP3: The NOD-like receptor family, pyrin domain-containing protein 3
PAMPs: Pathogen-associated molecular patterns
PRRs: Pattern recognition receptors
ROS: Reactive oxygen species
ROSC: Return of spontaneous circulation
