# Supplementary material for: MCC950, a selective NLPR3 inflammasome inhibitor, improves neurologic function and survival after cardiac arrest and resuscitation
Source: J Neuroinflammation. 2020 Aug 31;17:256. doi: 10.1186/s12974-020-01933-y (PMC7457538; doi:10.1186/s12974-020-01933-y)
Supplement: Supplementary file 1 — Additional file 1: Figure S1. Long-term outcome after CA/CPR in MCC950-treated mice. Supplemental data to Fig. 3. Mice were subjected to sham or CA/CPR surgery. After 15 minutes reperfusion, mice received intraperitoneal injection of MCC950 (10 mg/kg) or vehicle, followed by daily injection for 2 days. Rotarod and neurologic scoring were performed (A) on day 7 and (B) on day 14. The object location memory test was performed (C) from day 10 to 13. A schematic (right) illustrates 3 trial phases in the object location memory test. Data are presented as mean ± SEM (n = 3-8/group). **, p < 0.05; **, p < 0.01; ***, p < 0.001. Figure S2. Flow cytometric analysis of immune cells in the post-CA brain. Supplemental data to Fig. 4. A) The main flow cytometric gating strategy for infiltrating monocytes/macrophages and neutrophils. B) Mice were subjected to sham or CA/CPR surgery. After 15 minutes reperfusion, mice received intraperitoneal injection of MCC950 or vehicle, followed by daily injection for 2 days. On day 3 after CA/CPR, brains were collected for flow cytometry. Quantification of infiltrating leukocyte subsets of T (CD45+hiCD3+), B (CD45+hiCD3-CD19+), and NK (CD45+hiCD3-NK1.1+) cells is shown. Data are presented as mean ± SEM (n = 5/group). Figure S3. Disruption of blood-brain barrier (BBB) is not obviously evidenced in the post-CA brain. Mice were subjected to 45 minutes middle cerebral artery occlusion (MCAO) or 8.5 minutes CA. Extravasation of Evans blue (blue color) was evaluated at 3 hours or 24 hours after reperfusion. While BBB breakdown was clearly shown in brains after MCAO, there was no obvious BBB damage in the post-CA brains, indicating a comparably minor effect of CA/CPR on BBB permeability in our CA/CPR model. Representative images are shown from 2 independent experiments. Figure S4. The main gating strategy for analyzing immune cell popoluations in the spleen. Table S1. Primer sequences. Table S2. Antibodies for Western blotting and flow cytometry anal [file 12974_2020_1933_MOESM1_ESM.pdf]

Supplemental data

MCC950, a selective NLPR3 inflammasome inhibitor, improves neurologic function and survival after cardiac arrest and resuscitation

Maorong Jiang[1,2,*]; Ran Li[1,*]; Jingjun Lyu[1,3]; Xuan Li[1]; Wei Wang[1]; Zhuoran Wang[1]; Huaxin Sheng[1]; Weiguo Zhang[4]; Jörn Karhausen[1]; Wei Yang[1]

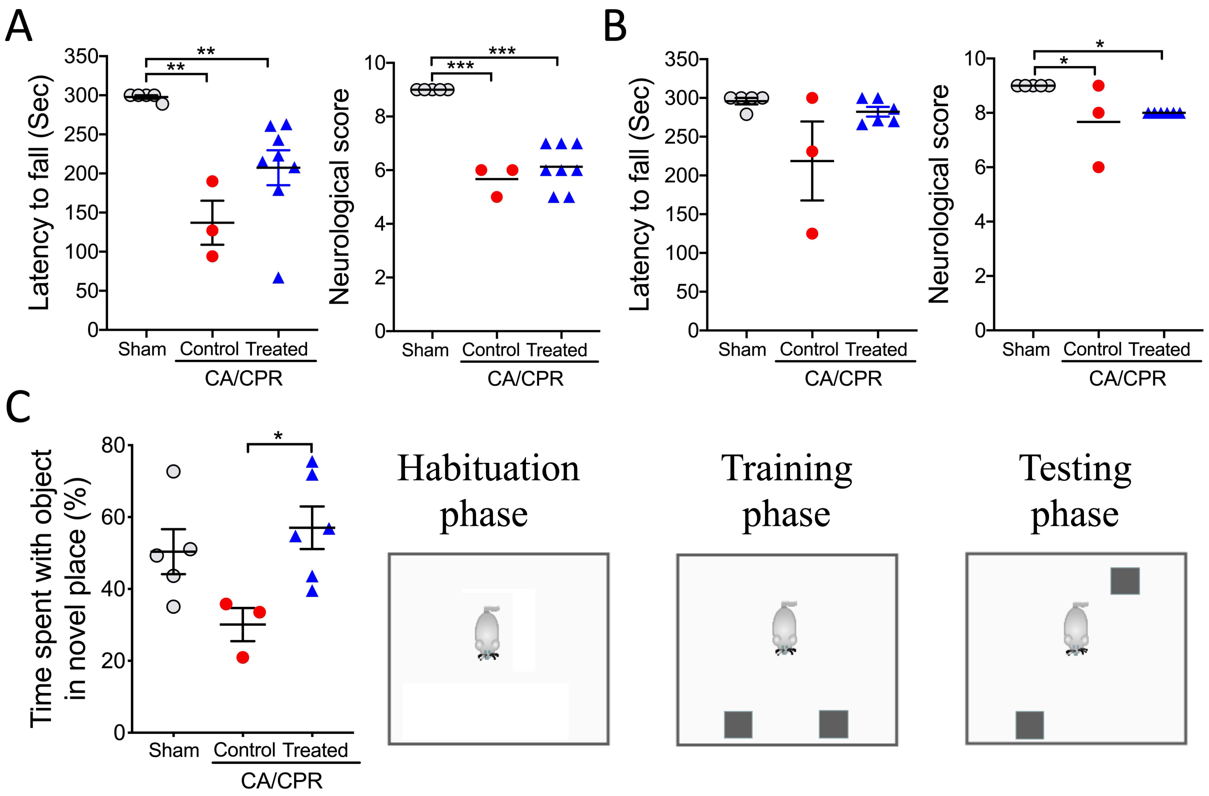

Figure S1. Long-term outcome after CA/CPR in MCC950-treated mice. Supplemental data to Fig. 3. Mice were subjected to sham or CA/CPR surgery. After 15 minutes reperfusion, mice received intraperitoneal injection of MCC950 (10 mg/kg) or vehicle, followed by daily injection for 2 days. Rotarod and neurologic scoring were performed (A) on day 7 and (B) on day 14. The object location memory test was performed (C) from day 10 to 13. A schematic (right) illustrates 3 trial phases in the object location memory test. Data are presented as mean ± SEM (n = 3-8/group). **, $p < 0.05$; **, $p < 0.01$; ***, $p < 0.001$.

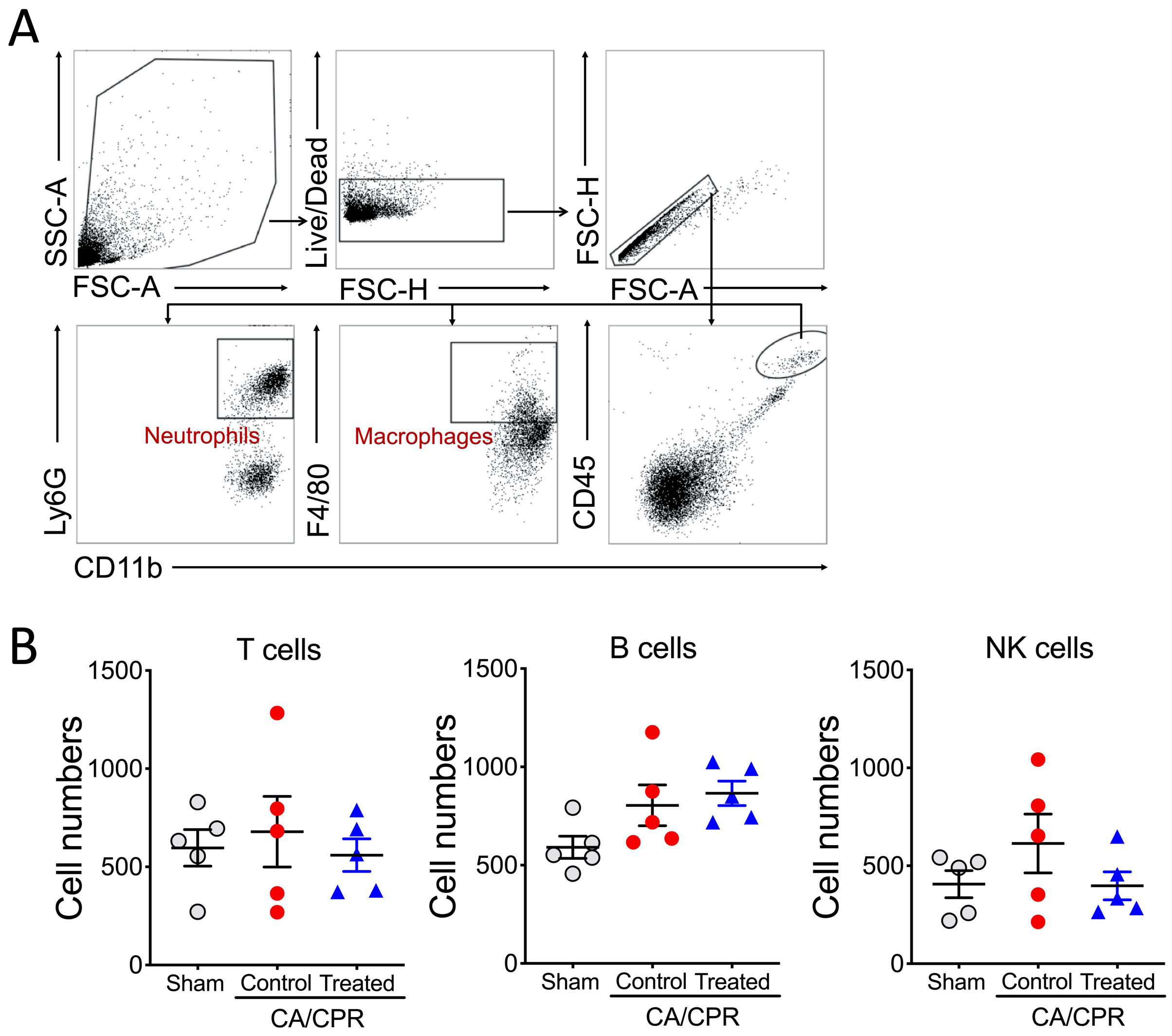

Figure S2. Flow cytometric analysis of immune cells in the post-CA brain. Supplemental data to Fig. 4. **A)** The main flow cytometric gating strategy for infiltrating monocytes/macrophages and neutrophils. **B)** Mice were subjected to sham or CA/CPR surgery. After 15 minutes reperfusion, mice received intraperitoneal injection of MCC950 or vehicle, followed by daily injection for 2 days. On day 3 after CA/CPR, brains were collected for flow cytometry. Quantification of infiltrating leukocyte subsets of T ($CD45^{+hi}CD3^{+}$), B ($CD45^{+hi}CD3^{-}CD19^{+}$), and NK ($CD45^{+hi}CD3^{-}NK1.1^{+}$) cells is shown. Data are presented as mean ± SEM (n = 5/group).

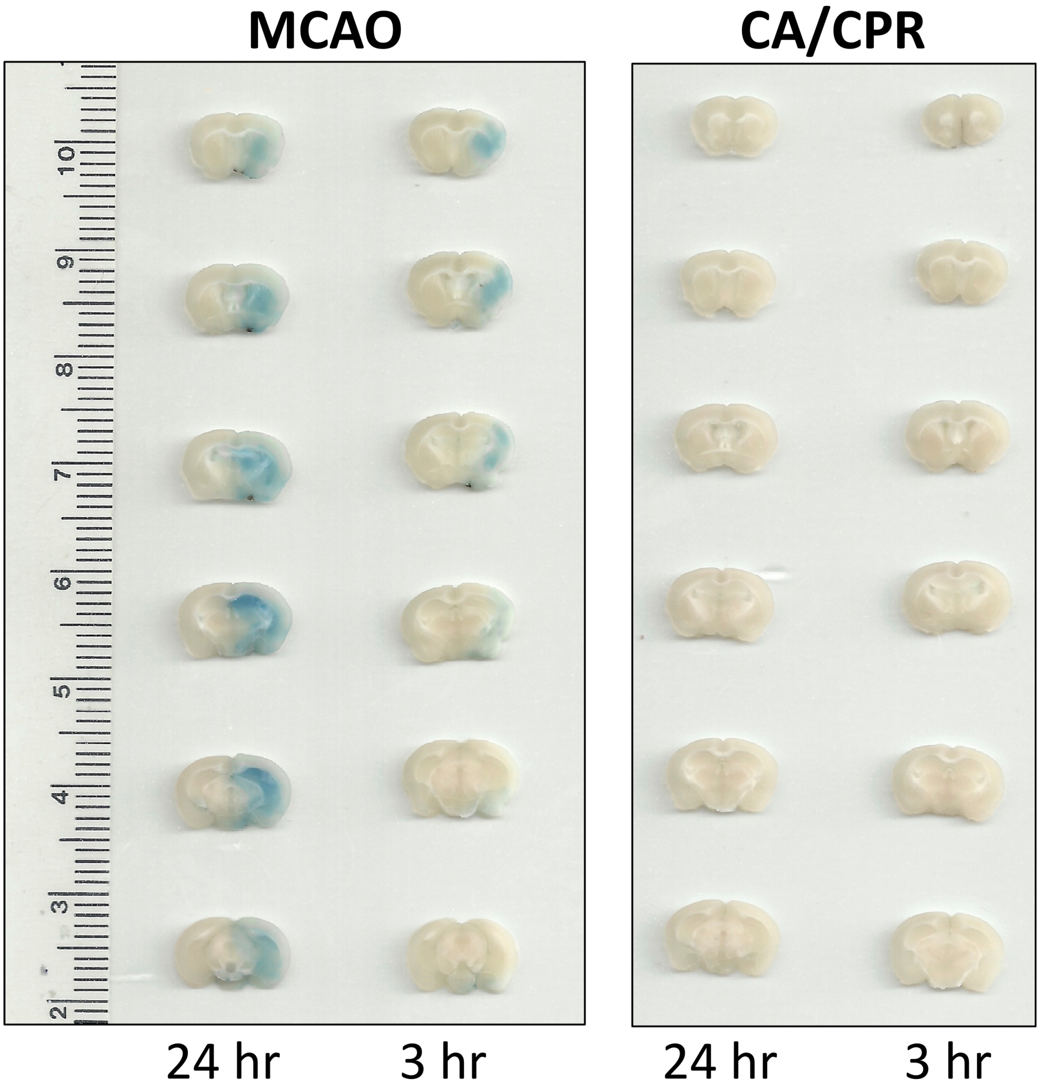

Figure S3. Disruption of blood-brain barrier (BBB) is not obviously evidenced in the post-CA brain. Mice were subjected to 45 minutes middle cerebral artery occlusion (MCAO) or 8.5 minutes CA. Extravasation of Evans blue (blue color) was evaluated at 3 hours or 24 hours after reperfusion. While BBB breakdown was clearly shown in brains after MCAO, there was no obvious BBB damage in the post-CA brains, indicating a comparably minor effect of CA/CPR on BBB permeability in our CA/CPR model. Representative images are shown from 2 independent experiments.

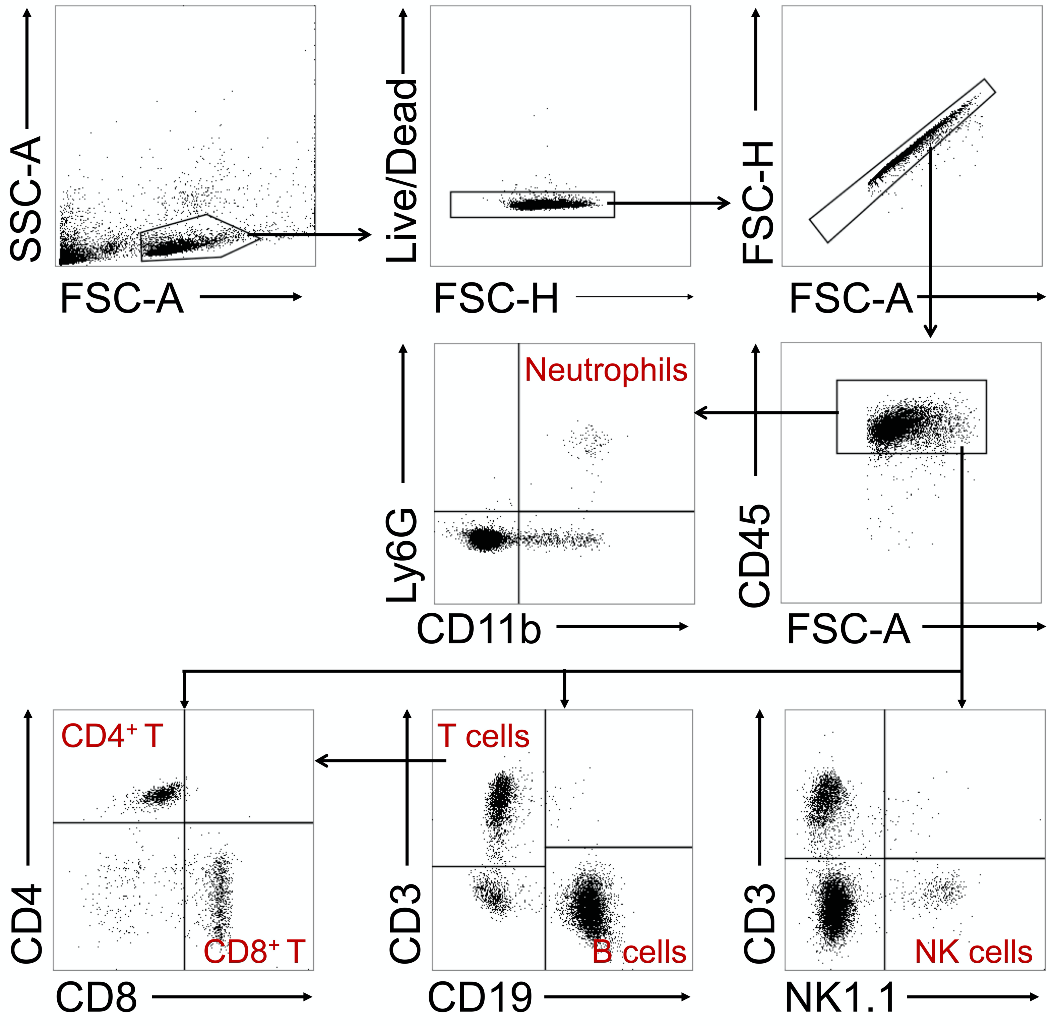

Figure S4. The main gating strategy for analyzing immune cell popoluations in the spleen.

Table S1. Primer sequences.

Gene		Primer sequence (5'-3')
TGF-β	Forward	GCAACATGTGGAACTCTACCAG
	Reverse	GTTGGTATCCAGGGCTCTCC
TNF-α	Forward	TGCCTATGTCTCAGCCTCTTC
	Reverse	CTCCTCCACTTGGTGGTTTG
IL-1β	Forward	CAGGCAGGCAGTATCACTCA
	Reverse	GCCCAAGGCCACAGGTAT
NLRP3	Forward	GCCCAAGGAGGAAGAAGAAG
	Reverse	AGGGCATTGTCACTGAGGTC
IL-18	Forward	GACTCTTGCGTCAACTTCAAGG
	Reverse	GGTCACAGCCAGTCCTCTTA
IL-10	Forward	AGCCGGGAAGACAATAACTG
	Reverse	TCACTCTTCACCTGCTCCAC
GAPDH	Forward	TGGAGAAACCTGCCAAGTATG
	Reverse	ATGTAGGCCATGAGGTCCAC

Table S2. Antibodies for Western blotting and flow cytometry analysis.

Applications	Antibody	Sources
Western bloting	NLRP3 (D4D8T)	Cell Signaling
	ASC (D2W8U)	Cell Signaling
	Cleaved caspase-1(E2G21)	Cell Signaling
	β-actin (A3854)	Sigma
Flow cytometry	CD45–FTIC (30-F11)	BioLegend
	CD3–APC (145-2C11)	BioLegend
	CD4–PE/Cy7 (GK1.5)	BioLegend
	CD8–APC/Cy7 (YTS156.7.7)	BioLegend
	NK1.1–PE (PK136)	BioLegend
	CD19–PE/Cy5 (6D5)	BioLegend
	CD45–PE (30-F11)	BioLegend
	F4/80–PE/Cy5 (BM8)	BioLegend
	Ly6G–FITC (1A8)	BioLegend
	CD11b–PE/Cy7 (M1/70)	BioLegend
